# Supplementary material for: Smooth Interpolating Curves with Local Control and Monotone Alternating Curvature
Source: Comput Graph Forum. 2022 Oct 6;41(5):25–38. doi: 10.1111/cgf.14600 (PMC9827861; doi:10.1111/cgf.14600)
Supplement: Supplementary file 1 — Supplement Material [file CGF-41-25-s001.zip › Local-Smooth-Interpolating-MonoCurvature/extern/clothoids/submodules/Utils/docs/api-cpp/define_a00029_1a144d062bf42ef80e2fb48e0ea0b9112e.html]

Define FMT\_ARGS — Utils v1.2

### Navigation

- index
- toc
- previous
- Utils »
- C++ API »
- Define FMT\_ARGS

# Define FMT\_ARGS¶

- Defined in File os.cc

## Define Documentation¶

Warning

doxygendefine: Cannot find define “FMT\_ARGS” in doxygen xml output for project “doc\_cpp” from directory: \_doxygen/doc\_cpp/xml-cpp

### Quick search

### Table of Contents

- rang - Colors for your Terminal
- Zstr
- C++ API

«
hide menu

menu
sidebar
»

### Navigation

- index
- toc
- previous
- Utils »
- C++ API »
- Define FMT\_ARGS

© Copyright 2021, Enrico Bertolazzi.
Created using Sphinx 4.2.0.
